# Graphene-Based Materials for Stem Cell Applications

**DOI:** 10.3390/ma8125481

**Published:** 2015-12-11

**Authors:** Tae-Hyung Kim, Taek Lee, Waleed A. El-Said, Jeong-Woo Choi

**Affiliations:** 1 School of Integrative Engineering, Chung-Ang University, 84 Heukseok-ro, Dongjak-gu, Seoul 06974, Korea; thkim0512@cau.ac.kr; 2 Interdisciplinary Program of Integrated Biotechnology, Sogang University, 35 Baekbeom-ro, Mapo-gu, Seoul 04107, Korea; tlee@sogang.ac.kr (T.L.); waleed@sogang.ac.kr (W.A.E.-S.); 3 Chemistry Department, Faculty of Science, Assiut University, Assiut 71516, Egypt; 4 Department of Chemical & Biomolecular Engineering, Sogang University, 35 Baekbeom-ro, Mapo-gu, Seoul 04107, Korea

**Keywords:** graphene, graphene oxide, graphene hybrid materials, graphene scaffolds, stem cells, differentiation, transplantation, detection, biomedical applications, stem cell engineering

## Abstract

Although graphene and its derivatives have been proven to be suitable for several biomedical applications such as for cancer therapy and biosensing, the use of graphene for stem cell research is a relatively new area that has only recently started to be investigated. For stem cell applications, graphene has been utilized by itself or in combination with other types of materials such as nanoparticles, nanofibers, and polymer scaffolds to take advantage of the several unique properties of graphene, such as the flexibility in size, shape, hydrophilicity, as well as its excellent biocompatibility. In this review, we will highlight a number of previous studies that have investigated the potential of graphene or its derivatives for stem cell applications, with a particular focus on guiding stem cell differentiation into specific lineages (e.g., osteogenesis, neurogenesis, and oligodendrogenesis), promoting stem cell growth, stem cell delivery/transplantation, and effective monitoring of their differentiation. We hope that this review promotes and accelerates the use of graphene-based materials for regenerative therapies, especially for stem cell-based approaches to cure various incurable diseases/disorders such as neurological diseases (e.g., Alzheimer’s disease and Parkinson’s disease), stroke, spinal cord injuries, bone/cartilage defects, and cardiovascular diseases.

## 1. Introduction

Graphene is a formation of carbon atoms in a two-dimensional honeycomb structure [1,2,3]. It has been intensively studied and is known to possess several unique properties such as high opacity (~97.7%), excellent electrical conduction ability (carrier mobility: 10000 cm^2^·V^−1^·s^−1^), and superior mechanical strength (Young’s modulus: 1100 GPa), which have propelled the utilization of graphene for electronic, electrochemical, and optical applications [4,5,6,7,8]. Besides the aforementioned characteristics, recent studies have also uncovered a number of fascinating properties of graphene, including high photoconversion efficiency, tunable amphiphilicity, excellent drug loading capacity, flexibility in size, and low cytotoxicity, all of which are useful features for nanomaterial-based biomedical applications such as cancer and stem cell therapies [9,10,11,12,13,14,15,16].

Compared to their use in cancer researches, the use of graphene-based materials in stem cell applications is a relatively new area of research [17,18,19,20,21,22,23]. As discussed in numerous research articles, stem cells have emerged as one of the most promising candidates for regenerative therapies because of their unique properties of self-renewal and differentiation. It is predicted that differentiated stem cells will be implantable in various tissues, which will ultimately be useful for the treatment of a wide range of diseases. Although promising, there are several issues that hinder the practicality of stem cells for regenerative therapies, including (1) low *ex vivo* differentiation efficiency; (2) low engraftment efficiency after transplantation; and (3) lack of the tools that would allow scientists and doctors to rapidly, easily, and precisely implant stem cells without sacrificing their viability and functionality [24,25,26,27,28,29,30].

The most common method for guiding the differentiation of stem cells involves the use of soluble factors including proteins, small molecules, and mixed supplements, all of which have to be carefully tuned based on the individual application [31,32]. In addition to the soluble cues, it has been reported that insoluble cues, which encompass the establishment and manipulation of extracellular microenvironments, especially the underlying substrates wherein cells attach and grow, have significant roles in controlling stem cell behaviors such as migration, proliferation, differentiation, and apoptosis [33,34,35,36,37,38,39,40]. Interestingly, graphene has proven to be capable of directing stem cell differentiation into specific cell types such as neurons, oligodendrocytes, osteoblasts, and adipocytes, based on the type of material (e.g., graphene, graphene oxide, and graphene hybrid scaffolds), as well as the type of progenitor cell (e.g., neural stem cells and mesenchymal stem cells) [41,42,43,44,45,46,47]. It can be used to tune such physical properties as elasticity, porosity, and micro/nano structure.

Besides the ability of graphene derivatives to guide differentiation of stem cells, graphene has shown an immense potential as an implantable material; it can help stabilize the growth and differentiation of stem cells embedded in three-dimensional hydrogels, thereby enhancing the efficiency of engraftment after transplantation [46,48,49]. Additionally, graphene has value as a detection molecule. Its surface absorbs specific molecules released from cells or embedded on cell membranes and enhances optical and/or electrical signals detectable by external analytical techniques [50]. Hence, despite a short history of utilization, considering the impact of stem cell-based regenerative therapies, it is worthwhile to summarize and highlight the recent progress of the use of graphene and/or graphene-based hybrid scaffolds for stem cell applications.

Therefore, in this review, we will discuss the biomedical applications of graphene-based materials, with a particular focus on guiding stem cell differentiation, stem cell transplantation/delivery, and monitoring/detection of stem cell differentiation. There are several review articles that discuss the use of graphene derivatives as therapeutic materials [51,52]. However, the utilization of graphene-based materials for stem cell applications is a rapidly emerging area and, thus, needs to be highlighted in order to fully understand their applicability and to envision the full potential of graphene in stem cell research.

## 2. Graphene and Graphene Oxide

Among the host of graphene derivatives, graphene and graphene oxide (GO) have been the most popular materials used for stem cell-based researches [53,54,55]. Graphene is composed of pure carbon atoms; however, graphene oxide contains many hydroxyl groups on its surface (–COOH, –C–O–C–, C–O–H, *etc.*), which makes it resistant to electron transfer. The controllable hydrophilic/hydrophobic nature of GO is one major difference between GO and graphene, and it leads to a difference in directing stem cell differentiation. A reduced graphene oxide (rGO) is another commonly used graphene derivative that is obtained from GO via chemical, thermal, or electrochemical reduction processes [56,57,58]. The rGO possess different surface characteristics (*i.e*., of a hydrophilic/hydrophobic nature) that are different from both graphene and GO and thus affect stem cell behaviors in a different manner. Still debatable, the toxicity of graphene derivatives has been intensively investigated for more than five years and, as shown in Table 1, they have turned out to be safe even at high concentrations [59,60,61,62,63,64,65]. In this subsection, we will discuss several strategies for guiding stem cell differentiation using different types of graphene derivatives and their potential for stem cell-based regenerative therapies.

**Table 1:** Toxicity evaluation of graphene and graphene oxide (GO).

Material	Model	Maximum Dose (μg/mL)	Safe Dose (μg/mL)	Reference
Graphene	Monkey renal cells	300	<50	[59]
GO	Neuroblastoma	100	<80	[60]
	Human retinal pigment epithelium cells	100	<100	[61]
	Red blood cells	75	<75	[62]
	Human fibroblast	100	<50	[63]
	Human alveolar basal epithelial cells	200	<200	[64]
	Mouse embryonic fibroblast	1000	<1000	[65]

### 2.1. Enhancing Osteogenesis with Graphene/Graphene Oxide

**Figure 1:**
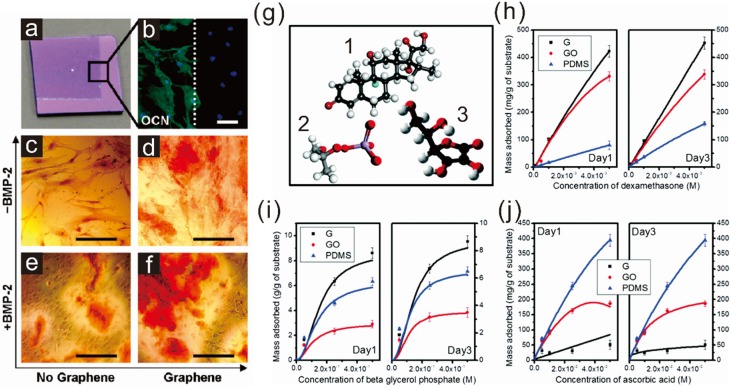
Acceleration of osteogenic differentiation of mesenchymal stem cells. (**a**) Image of partially graphene-coated Si/SiO_2_ substrate; (**b**) Cells on graphene showing enhanced expression of one of the representative osteogenic marker, osteocalcin (OCN); (**c**–**f**) Enhanced calcifications of cells on graphene-coated substrate confirmed by alizarin red staining. Reprinted with permission from [41]. Copyright 2011 American Chemical Society. Scale bars = 100 μm; (**g**) Chemical structures of three different molecules responsible for osteogenic differentiation: (**1**) dexamethasone, (**2**) beta glycerol phosphate anion, and (**3**) ascorbic acid; (**h**–**j**) Loading capacity of (**1**–**3**) on graphene (G), graphene oxide (GO), and PDMS. Reprinted with permission from [42]. Copyright 2011 American Chemical Society.

The first study investigating the potential of graphene in guiding stem cell differentiation was reported by Nayak *et al.* in 2011 [41]. The authors utilized a graphene-coated silicon wafer fabricated by chemical vapor deposition (CVD), a method proven to be highly efficient in generating high-quality graphene, to guide osteogenesis of bone marrow-derived mesenchymal stem cells (bmMSCs). Mesenchymal stem cells are one of the major progenitor cell lines achievable from a number of sources including adipose tissues, bone marrow, embryonic stem cells (ESCs), and induced pluripotent stem cells (iPSCs). They are capable of generating multiple cell types such as fibroblasts, myoblasts, adipocytes, chondrocytes, and osteoblasts, all of which are important for the regeneration of damaged tissues in the human body. Interestingly, graphene was found to significantly accelerate and enhance osteogenesis of bmMSCs, regardless of the type of underlying substrate (such as glass slide, Si/SiO_2_ wafer, polyethylene terephthalate (PET), and polydimethylsiloxane (PDMS)), based on the level of osteocalcin (a marker for osteogenesis) and calcium mineralization level (Figure 1a–f). The reason for enhanced stem cell differentiation to an osteogenic lineage was explained by Lee *et al.* in 2011 [42]. They studied the capability of graphene and GO to absorb surface factors including proteins (insulin) and small molecules (dexamethasone, beta glycerol phosphate, and ascorbic acid), which are the essential components for adipogenic and osteogenic differentiation of MSCs (Figure 1g–j). GO showed the best absorption of proteins because of a strong electrostatic interaction between oxygenated groups present on its surface and components of the protein. Interestingly, graphene was also found to be effective in attracting protein components; PDMS, which is also highly hydrophobic, shows a very low level of protein absorption. It is thought that this is due in part to the π-electron cloud in graphene, which is capable of interacting with hydrophobic protein cores. Osteogenesis-related small molecules showed different absorption kinetics towards graphene, GO, and PDMS. Both graphene and GO were found to effectively attract dexamethasone; however, GO did not adhere to beta glycerol phosphate, while graphene strongly adhered to that molecule. Ascorbic acid (Vitamin C), which is important for several stem cell differentiation processes (e.g., neurogenesis, chondrogenesis, and osteogenesis), was more stably absorbed on the surface of GO than was graphene. Later on, Alzhavan *et al.* also reported a graphene nanogrid that was effective for selective and fast osteogenic differentiation of human mensenchymal stem cells (hMSCs) [66]. Here, the authors utilized graphene obtained from graphene nanoribbons through the unzipping of carbon nanotubes. The rGO ribbon grid showed accelerated osteogenic differentiation of the hMSCs with enhanced differentiation efficiency that was 2.2-fold greater than rGO film. Hence, we conclude that both graphene and GO are suitable materials for enhancing bone cell regeneration from MSCs. Graphene was more effective than GO in the presence of osteogenic media.

### 2.2. Enhancing Neurogenesis by Graphene/Graphene Oxide

Neural stem cells (NSCs), which are capable of generating different types of neuronal cells such as neurons, oligodendrocytes, and astrocytes, are also possible candidates for interference by nanomaterials. It has been reported that hNSCs tend to generate more glial cells, especially astrocytes, than neurons in natural states, even though neurons are largely considered to be more critical than glial cells for the treatment of neurological diseases/disorders [67,68,69]. Hence, directing differentiation of hNSCs into neurons has been a major interest in the field of stem cell-based regenerative medicine. Interestingly, in 2011, Park *et al.* investigated the effects of graphene on differentiation of hNSCs and found that graphene coated with an extracellular matrix (ECM) protein essential for the adhesion of neuronal cells, laminin, was useful in cellular support [45]. Interestingly, the authors reported that hNSCs rapidly accumulated on the graphene-coated surface after 10 h of incubation, which indicates that neuronal cells may prefer graphene-modified surfaces for their growth.

**Figure 2:**
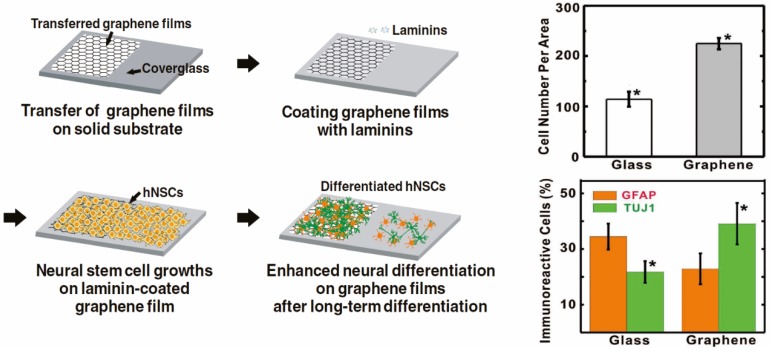
The enhancement of growth and differentiation of human neural stem cells (hNSCs) on graphene-coated cover glass. β3 Tubulin (Tuj1) and glial fibrillary acidic protein (GFAP) are the representative markers of neurons and glial cells, respectively. Reprinted with permission from [45]. Copyright 2011 John Wiley and Sons.

This graphene-induced difference in cell adhesion and growth was found to be completely negligible when cells were cultured for more than five days. When cells were treated with differentiation induction medium, a graphene-coated surface produced better cell adhesion, resulting in increased cell numbers, cellular growth, and differentiation toward neuronal lineages that was confirmed by two representative markers of glial [Glial fibrillary acidic protein (GFAP)] and neuronal [β3 Tubulin (Tuj1)] cells (Figure 2). Later on Akhavan *et al.* reported a graphene-based approach to direct differentiation of hNSCs into neurons [70]. In this study, the reduced graphene oxide nanoribbon (rGONR) grids, in conjunction with photocatalytic stimulation, were found to significantly enhance neuronal network formation when compared to normal quartz substrates after three weeks of differentiation. Additionally, GO was also found to promote dopaminergic differentiation of mouse embryonic stem cells (mESCs) [71]. Interestingly, among many different carbon-based materials including CNTs, GO and graphene, only GO was found to be effective in guiding the generation of dopaminergic neurons from mESCs. While they are still promising, one major hurdle in the use of hNSCs for regenerative therapies is that hNSCs are generally located in deep brain tissues, and therefore inaccessible. Hence, certain types of stem cells, such as bone marrow- or adipose tissue-derived MSCs, which are convenient, safe, and easy to obtain with minimal patient discomfort, are good options in the treatment of neurological diseases/disorders. However, the conversion of MSCs to functional neuronal cells is highly challenging since it necessitates germline transition from ectodermal to mesodermal cells, which is difficult to achieve using conventional differentiation methods. Interestingly, Kim *et al.* recently reported a potential applicability of graphene to this process [44]. The authors utilized a highly uniform graphene generated by sequential CVD on PMMA-covered copper foil and transferred the CVD graphene to a glass cover slip. Remarkably, MSCs grown on graphene-coated substrates showed a higher expression level of several important neuronal cell markers, such as Nestin, Tuj1 and Map2, as confirmed by qRT-PCR, with or without neurogenic induction media. Therefore, it can be concluded that graphene itself, without any chemical and/or physical modifications, holds huge potential as an effective nanomaterial for directing the differentiation of stem cells; that characteristic may very well influence the practical use of stem cells in regenerative medicine.

## 3. Graphene-Based Scaffold Materials

Since graphene is a flexible sheet-like material, it can be easily modified on the surface of polymeric structures, allowing for the unique physicochemical properties of graphene derivatives while maintaining their topographic features. Besides graphene-polymer hybrid scaffolds, interestingly, graphene itself was reported to be able to form complex three-dimensional structures that are suitable for long-term cell growth without showing cytotoxicity and other adverse effects. Recently, graphene was also found to be useful as a cellular adhesive that is effective in preventing implanted cells from reactive oxygen species (ROS)-mediated cell death, thereby enhancing the therapeutic efficiency of MSCs. In this subsection, we will discuss several applications of graphene or graphene hybrid scaffolds used in various stem cell applications, such as stem cell differentiation, delivery, and transplantation.

### 3.1. Graphene Polymer Scaffolds for Guiding Stem Cell Differentiation

Polymer scaffolds are highly desirable cell-supporting materials because of their flexibility in various aspects including size, shape, mechanical properties, surface functionalities, structural formations, and biocompatibility. Among the large number of polymer materials available, electrospun nanofiber scaffolds, which are normally generated by electrical force applied between metallic collectors and spinning tips, are one of the most widely used polymer constructs for cell/tissue engineering because of their structural formation that mimics natural ECM scaffolds. Gelatin, collagen, polycaprolactone (PCL), and poly(lactic-co-glycolic acid) (PLGA) have been widely utilized to generate electrospun nanofibers because they are superior in biodegradability and biocompatibility [72,73,74,75].

**Figure 3:**
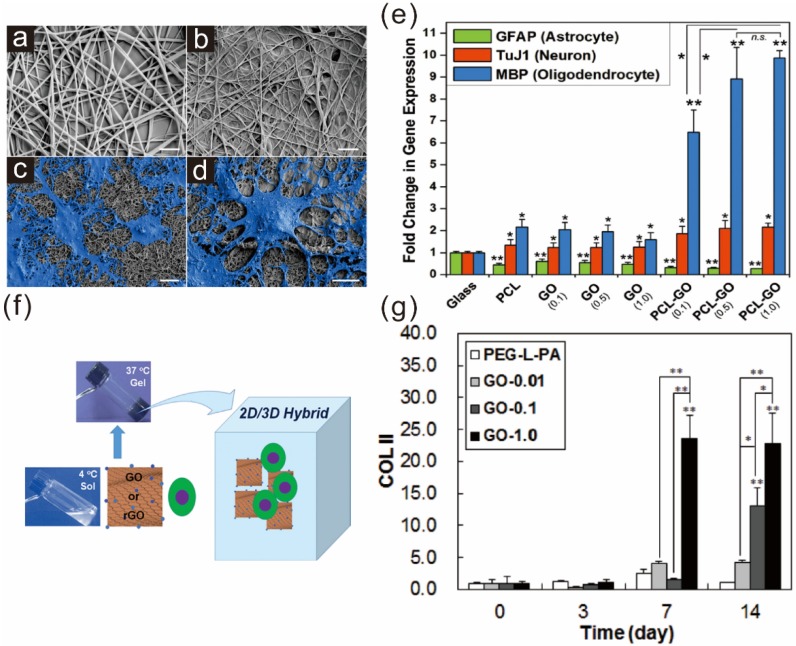
Graphene hybrid scaffolds enhancing stem cell differentiation into specific lineages: (**a**) nanofiber scaffold; (**b**) graphene-nanofiber hybrid scaffold; and (**c**–**d**) differentiated neural stem cells on graphene-PCL nanofiber scaffolds. Scale bars = 10 μm; (**e**) Quantitative analysis of several markers such as GFAP (astrocyte), Tuj1 (neuron), and MBP (oligodendrocyte). GO-modified PCL nanofibers showed the highest expression of MBP. Student’s unpaired t-test was used for calculating significance (* *p* < 0.05, ** *p* < 0.01, n.s. = no significance). Reprinted with permission from [46]. Copyright 2014 John Wiley and Sons; (**f**) Schematic diagram depicting the preparation of the 2D/3D hybrid cell culture platform; (**g**) Enhancement of chondrogenic marker (COLII) during the TMSCs culture in GO-containing polymer scaffolds. Reprinted with permission from [48]. Copyright 2014 John Wiley and Sons.

Recently, Shah *et al.* showed the effects of GO-modified PCL nanofibers on the differentiation of neural stem cells [46] (Figure 3a–e). In this study, GO was directly deposited on the surface of an oxygen plasma-treated PCL polymer scaffold, followed by ECM protein (laminin) absorption, essential for the adhesion, growth, and differentiation of NSCs. Remarkably, the GO-functionalized PCL nanofibers were shown to enhance oligodendrogenesis of NSCs, which was confirmed by several early oligodendrocyte markers (e.g., CNP, PDGFR, Olig1 and Olig2) and mature oligodendrocyte markers (e.g., PLP, MBP, MAG and MOG). The enhancement of oligodendrogenesis of NSCs on PCL-GO nanofiber scaffolds was found to be linked to several integrin-related key signaling proteins including focal adhesion kinase (FAK), integrin-linked kinase (ILK), Akt, and Fyn kinase (Fyn). Since oligodendrocytes are known to be important for the regeneration of damaged parts of neuronal networks, the authors claimed that the GO-PCL hybrid scaffolds will be highly beneficial for developing future substrate-based therapies for central nervous system (CNS)-related injuries, diseases, or disorders.

In addition to the effectiveness in two-dimensional culture platforms, GO was also reported to be suitable in generating 2D/3D hybrid cell culture systems when combined with temperature-responsive biocompatible polymers [48] (Figure 3f). One example is a hydrogel composed of poly(ethylene glycol)-poly(L-alanine) diblock copolymer (PEG-L-PA), GO/rGO, and tonsil tissue-derived mesenchymal stem cells (TSMCs), wherein GO or rGO is used for the purpose of encapsulating TSMCs within hydrogel scaffolds. Interestingly, TSMCs cultured in an rGO-embedded hybrid culture system showed a 30%–50% increase in proliferation when compared with GO/PEG-L-PA hybrid scaffolds, while both GO and rGO hybrid systems were proven to maintain the spherical morphology of TSMCs. In contrast, in terms of chondrogenic differentiation, polymer-GO hybrid scaffolds were found to be better than rGO hybrid cell culture platforms when treated with TGF-β3-containing medium, and showed enhanced expression of several chondrogenic markers such as SOX 9, COL 2A1, COL 2, and COL X (Figure 3g). Since chondrogenic differentiation of MSCs is extremely important for the treatment of chondral or osteochondral damages, the use of graphene hydrogels to enhance chondrogenesis of MSCs is an attractive strategy for treatment.

### 3.2. Graphene as a Biocompatible Material for Stable Stem Cell Growth and Implantation

Besides the ability of graphene derivatives (GDs) to steer stem cell differentiation, owing to their distinct physical and chemical characteristics, GDs are able to protect stem cells and provide microenvironments suitable for long-term *in vivo* survival, both of which are critical for successful stem cell transplantations. Park *et al.* recently reported a possible strategy of using GOs to prevent. 

**Figure 4:**
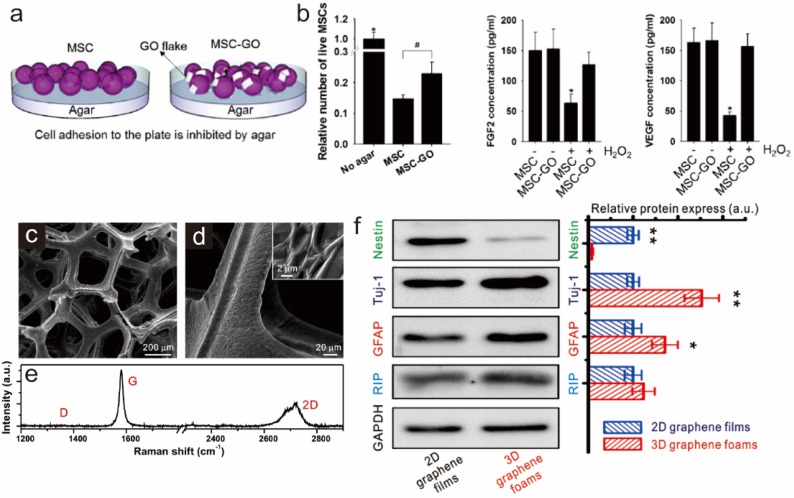
Protection of ROS-mediated cell death via graphene oxide (GO). (**a**) Viability of mesenchymal stem cells (MSCs) cultured on an agar plate with or without GO addition; (**b**) Amounts of two different proteins (FGF2 and VEGF) secreted by MSCs and GO-protected MSCs with or without hydrogen peroxide (H_2_O_2_). * *p* < 0.05 compared to any group; # *p* < 0.05; *n* = 3. Reprinted with permission from [49]. Copyright 2015 American Chemical Society; (**c**–**d**) SEM images of 3D graphene foams (3D-GFs); and (**e**) its Raman spectrum. Enhanced differentiation of neural stem cells cultured on 3D-GFs confirmed by western blot analysis. * *p* < 0.05, ** *p* < 0.01; (**f**) Western blot analysis of Nestin, Tuj-1, GFAP and RIP protein expression of differentiated NSCs on 2D graphene films and 3D-GFs. The Data are presented as mean ± standard error (s. e. m.), * *p* < 0.05, ** *p* < 0.01. Reprinted by permission from Macmillan Publishers Ltd.: Scientific Reports ([47]). Copyright 2013.

ROS mediated death of implanted cells, which could be effective for cardiac repair [49] (Figure 4a,b). The use of MSCs to treat myocardial infarction is normally hindered by generation of ROS in the ischemic myocardium after the restoration of blood flow. However, when GO was mixed with MSCs prior to the injection, GO was found to be absorbed on extracellular matrix (ECM) proteins, resulting in protection of implemented MSCs from ROS-mediated damages or cell death. The ability of GO to protect MSCs from ROS species was also further confirmed by *in vitro* experiments wherein hydrogen peroxide (H_2_O_2_) was used as a ROS-generator to damage MSCs. Several indicators of cell function, such as cell adhesion, viability, and protein secretion (e.g., FGF2 and VEGF), were all enhanced in MSCs mixed with GO in the presence of H_2_O_2_, indicating that GO has an exceptional capability to protect MSCs from ROS, even in harsh conditions. As expected, when cells were delivered to infarcted and reperfused myocardium with GO layer protection, cells showed successful secretion of reparative paracrine factors and decreased apoptosis of cardiac tissue, which in turn improved cardiac function.

In addition to cell encapsulation using GO, Sung *et al.* reported several studies in which three-dimensional structures composed of graphene and nickel template, termed graphene foams (GFs), were generated; GFs are biocompatible and suitable for stem cell growth [47,76] (Figure 4c–e). Interestingly, GFs were found to induce elongated morphologies of MSCs and enhance osteogenesis, which was consistent with previous reports utilizing graphene as a cell differentiation-controlling material, especially for osteogenesis on two-dimensional substrates. The authors also reported that GFs have the potential to culture NSCs: GFs were used as a three-dimensional cell supporting material to enhance cell growth and to facilitate the neurogenesis and astrocytogenesis of NSCs (Figure 4f). Owing to the excellent electrical properties of graphene, GFs allowed for electrical stimulation on differentiated neuronal cells, resulting in changes in intracellular calcium ion concentrations, which were confirmed by fluorescence imaging using Fluo-4 acetoxymethyl ester (Fluo-4 AM) dye. Besides graphene-modified porous composite materials, graphene was reported to be able to generate 3D free-standing porous scaffolds without incorporating any other supporting materials [77].

Serrano *et al.* reported this 3D scaffold structure purely composed of graphene, which allowed for stable growth of embryonic neural progenitor cells, with exceptional ability to generate interconnected neural networks including both neurons and glial cells having large number of dendrites, axons, and synaptic connections. Hence, it can be concluded that graphene or graphene-cell hybrid scaffolds are excellent materials for stable stem cell growth, spreading and differentiation *in vitro* and *in vivo*.

## 4. Graphene Patterns

Recently, the control of stem cell fate by modulating biophysical cues (e.g., micropatterns, nanopatterns, elasticity and porosity of the substrates) has emerged as an attractive stem cell-based therapy [33,37,78,79]. Interestingly, stem cells were found to have the ability to sense changes in their microenvironment, resulting in controlled differentiation via cell-substrate interactions that alter upstream cytoskeletal dynamics and downstream gene expression. Among the many different strategies available for the modulation of stem cell behavior, micropatterns (the periodic structures having specific shapes and sizes) have been reported to be highly efficient for controlling stem cell differentiation by restricting cell attachment, spreading, and morphology. Many different cell lineages have been reportedly acquired from stem cells whose differentiation lineages were controlled by micropatterning technologies [36,37,39,80,81]. The challenge, however, is the instability of ECM proteins, which control cell morphological behaviors through cell-substrate interactions, as well as the chemical linkers, which are essential for the absorption of ECM proteins and must be varied with different types of substrates. As a result, this instability compromises the tremendous potential of combinatorial hybrid pattern-based stem cell applications.

**Figure 5:**
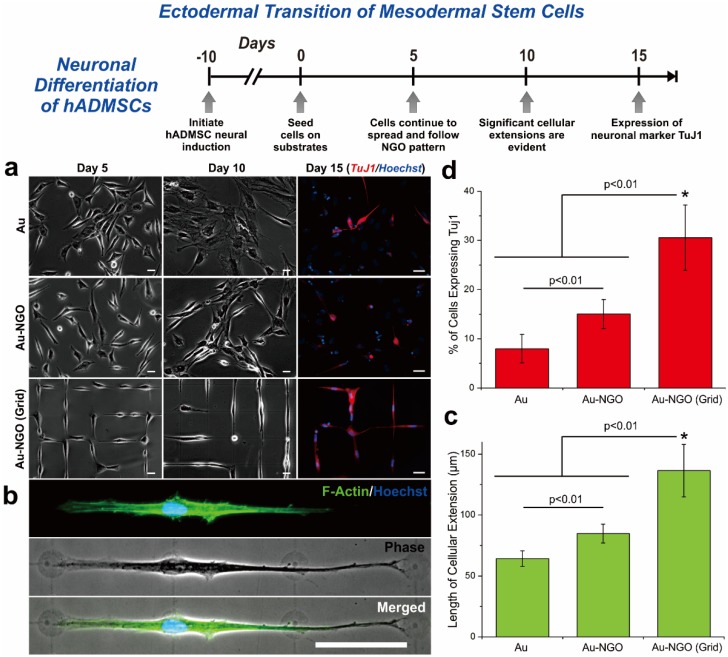
Neuronal differentiation of hADMSCs using NGO grid-patterned substrate. (**a**) Pictures of hADMSCs cultured on PLL-coated Au [Au], NGO-coated Au [Au-NGO], and NGO grid-patterned substrates [Au-NGO (Grid)]. Differentiation of hADMSCs confirmed by the neuronal marker TuJ1 (red) and nucleus (blue). Scale bars = 20 μm; (**b**) Optical and fluorescence images of hADMSCs stained for F-actin (green) and nucleus (blue). Scale bar = 50 μm; (**c**) Quantitative analysis of the length of cellular extension on various substrates (*n* = 3; * *p* < 0.01, Student’s unpaired t test); (**d**) Quantitative analysis of the percentage of cell showing the neuronal marker TuJ1 on various substrates (*n* = 3; * *p* < 0.01, Student’s unpaired t test). Reprinted with permission from [82]. Copyright 2015 American Chemical Society.

In 2015, Kim *et al.* reported an interesting study utilizing graphene oxide as a patterning material to control stem cell morphologies, to ultimately steer their differentiation into specific cell types such as osteoblasts and neurons [82]. Because of the physical characteristics of GO used in this study, which were small in size (100 nm) and highly hydrophilic (highly oxidized), GO was found to be effectively patterned on any kind of biocompatible substrate including conventional tissue culture plates (TCPs), gold-coated glass, flexible PDMS, and biodegradable polymer (PLGA) in different shapes and sizes. Remarkably, the generated GO patterns were highly stable for a long period of time even in harsh conditions, which may provide a possible solution to the problem of instability of protein-based micropatterns. The GO line patterns, in combination with cell-repulsive molecules, which mimic the terminal morphology of elongated differentiated osteoblasts, showed highly efficient osteogenic differentiation of human adipose-derived mesenchymal stem cells (hADMSCs) that were 54.5% and 41% higher than bare gold and GO-coated substrates, respectively. The GO-laminin hybrid patterns, which mimic the shape of interconnected neuronal network, were also reported to be effective to convert hADMSCs into neuron-like cells that were up to 30% higher than the control group (Figure 5). Since the neuronal differentiation of hADMSCs necessitates a germ layer transition that is highly challenging, the enhanced efficiency of neurogenesis of hADMSCs is quite remarkable, and could be useful in curing a number of existing neurological diseases and disorders such as Alzheimer’s disease, Parkinson’s disease, stroke and spinal cord injuries.

In addition to this study, Bashir *et al.* also recently investigated the effects of graphene patterns, which mimic the elongated structure of muscle cells, on the differentiation of skeletal muscle myoblasts (C2C12) [83]. The C2C12 cells grown on graphene line patterns followed geometric features of graphene patterns and, as a result, the differentiation of C2C12 was enhanced significantly compared to control groups.

The clear advantage of using graphene-based patterns is their ability to modulate stem cell morphologies, resulting in enhanced differentiation of mesenchymal stem cells into two different lineages (bone and neuronal cells), based on the types of patterns utilized (line and grid patterns). Additionally, compared to protein micropatterns to guide stem cell morphologies, graphene/GO is highly stable for a long period of time, which is important for affecting cells during a differentiation process that normally takes more than two weeks. Thus, it can be concluded that besides graphene-coated substrates and 2D or 3D graphene hybrid scaffold materials, graphene- or GO-patterned substrates could be highly effective in guiding the differentiation of many different types of stem cells into various lineages, such as osteoblasts, neurons, and myocytes.

## 5. Graphene-Based Hybrid Nanoparticles

There have been a number of interesting studies investigating the potential of graphene-based hybrid nanoparticles in biomedical applications such as biosensors, biochips, and stem/cancer therapies [84]. Graphene polymer nanoparticles, nanoparticle-decorated graphene sheets, graphene-embedded nanoparticles, and graphene-encapsulated nanoparticles are the forms most useful for biomedical applications. Of the many different types of graphene hybrid nanoparticles, in this subsection, we will highlight graphene-embedded and graphene-encapsulated nanoparticles and will discuss how these materials affect stem cell behaviors including cell growth and differentiation. Additionally, we will highlight interesting ways to utilize graphene-based hybrid nanoparticles to monitor differentiation of neural stem cells in a non-destructive and non-invasive manner, using surface-enhanced Raman spectroscopy as a detection tool.

### 5.1. Graphene-Embedded Hybrid Nanoparticles for Guiding Stem Cell Growth

**Figure 6:**
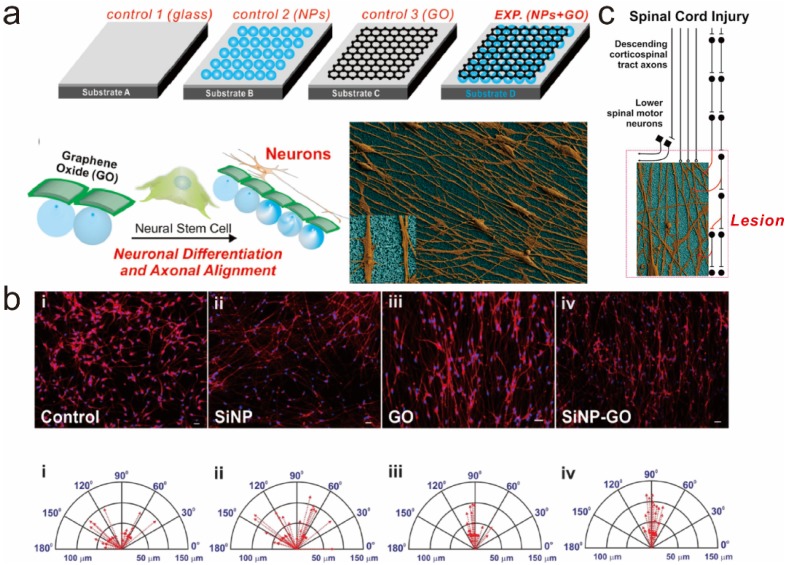
(**a**) Schematic diagram representing the effects of GO-coated nanoparticle hybrid construct on the differentiation of hNSCs and the alignment of the axons of differentiated neurons; (**b**) Comparison of aligned growth of differentiated hNSCs on different substrates [(**i**) Glass, (**ii**) SiNP-modified glass, (**iii**) GO-modified glass and (**iv**) SiNP-GO-modified glass]. Scale bars = 10 μm; (**c**) Possible strategy for utilizing the GO-coated nanoparticle hybrid construct (SiNP–GO) to treat spinal cord injury. Reprinted with permission from [85]. Copyright 2013 John Wiley and Sons.

In 2013, Solanki *et al.* reported a study investigating the effects of graphene-embedded nanoparticle hybrid structures on stem cell growth [85]. In this study, GO was encapsulated on the surface of positively-charged silica nanoparticles (SiNP-GO) via electrostatic interaction and then GO-encapsulated nanoparticles were packed on the surface of typical glass substrates. To allow cells to grow on the substrate, ECM protein (laminin) was further absorbed on the surface of GO prior to cell cultivation (Figure 6a). The fabricated hybrid nanoparticle-modified substrate was biocompatible, producing approximately 20% higher cell viability than both glass and SiNP-decorated glass substrates. Interestingly, the authors claimed that cells tended to be aligned on the SiNP-GO as they differentiate into the neuronal cells, which was evident compared to cells grown on other substrates (glass, SiNP-coated, and GO-coated), based on actin-stained fluorescence images and scanning electron microscopic (SEM) images (Figure 6b). SiNPs were previously shown to be effective in increasing the lengths of extending axons; SiNP-GO was also found to increase the lengths of extending axons, which were 20.76% and 11.3% higher than those cultured on glass and GO-coated substrates, respectively. In addition to cell alignment and extended axonal growth, interestingly, both SiNP-GO-coated glass and flexible PDMS substrates were shown to enhance the neuronal differentiation of NSCs, which was confirmed by several neuronal markers such as Tuj1, GAP43, MAP2, and synapsin. GO was better than graphene because of its many different polar groups (e.g*.*, epoxide, hydroxyl and carboxyl groups), which facilitate immobilization of GO on the surface of NPs via electrostatic charge and also contribute to rapid absorption of laminin, which is critical for stable cell growth and differentiation. Interestingly, when other two-dimensional materials, such as molybdenum disulfide (MoS2), which has a structure is similar to that of graphene, were functionalized on the surface of NPs, axonal alignment was not observed in neuronal cells derived from NSCs. Since both axonal guidance and neuronal differentiation are very important in the treatment of spinal cord injuries, in this study, the authors suggested an interesting strategy to utilize SiNP-GO substrate in the transplantation process, wherein the implanted substrate helps the regeneration and reconnection of neuronal circuits (Figure 6c).

### 5.2. Graphene Hybrid Gold Nanoparticles for Monitoring Stem Cell Differentiation

**Figure 7:**
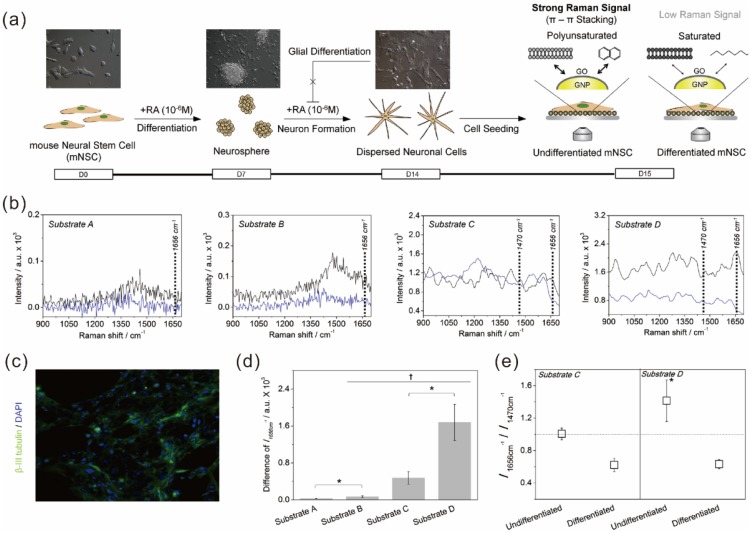
(**a**) Schematic diagram depicting the method to distinguish the undifferentiated and differentiated state of mouse neural stem cells (mNSCs) using 3D GO-encapsulated gold nanoparticles; (**b**) Raman signals achieved from undifferentiated (black) or differentiated (blue) mNSCs on different substrates including indium tin oxide (ITO, Substrate A), GO-modified ITO (Substrate B), gold nanoparticle-modified ITO (Substrate C), and ITO modified with GO-encapsulated gold nanoparticles (Substrate D); (**c**) Fluorescence images of differentiated mNSCs on Substrate D proving the successful differentiation of mNSCs to neurons; (**d**) Intensities of Raman peak (1656 cm^−1^) obtained from undifferentiated mNSCs subtracted by differentiated cells; (**e**) Relative Raman intensities at 1656 cm^−1^ divided by the intensities at 1470 cm^−1^. * *p* < 0.05, Student’s t-test. Reprinted from [50], Copyright 2013, with permission from Elsevier.

The potential of graphene hybrid nanoparticles is not restricted to the control of stem cell behaviors such as cell growth, adhesion, spreading, migration, and differentiation. As previously mentioned, besides controlling stem cell functions and behaviors, monitoring their differentiation process is a critical step for the practical use of stem cells in regenerative therapies. However, to identify terminal states of stem cells precisely, destructive steps such as cell lysis or fixation are essential for conventional analytical techniques including immunostaining, polymerase chain reaction (PCR) and/or biological tools including southern/northern/Western blotting. Addressing this issue, in 2013, Kim *et al.* reported a new method that enabled non-destructive *in situ* monitoring of stem cell differentiation [50]. They utilized a graphene-encapsulated gold nanoparticle (GO-GNPs) as a core material to induce surface-enhanced Raman scattering (SERS). To generate GO-GNP hybrid material, in this study, the GNPs were first functionalized with cysteamine to produce positive charges on their surface, and then the cysteamine-modified GNPs were further reacted with GO, resulting in formation of a stable GO shell on the surface of GNPs via electrostatic interaction. Interestingly, when GO–GNPs were immobilized on the surface of indium tin oxide (ITO), undifferentiated stem cells showed higher SERS signals because undifferentiated stem cells possess higher numbers of polyunsaturated membrane molecules than do differentiated cells. Since the unsaturated molecules, which generally contain benzyl groups, are highly adhesive on the surface of graphene and GO due to strong π–π interactions, it was hypothesized that the difference in the number of polyunsaturated molecules resulted in a difference in adhesion force between the GO–GNPs and the molecules, which in turn produced changes in Raman signals between undifferentiated and differentiated stem cells (Figure 7). This hypothesis was confirmed by analyzing Raman peaks obtained from two different molecules having different number of C=C bonds (Cidofovir and hydroxybenzoate) that exist in stem cells. The Raman signal corresponding to the C=C bond was highly enhanced on GO-GNP substrate compared to the normal GNP-modified substrate when analyzing hydroxybenzoate, while Cidofovir containing one C=C bond failed to show any difference in Raman signals between GNP–GO and GNP-modified substrates. It is important to note that the GNPs were incorporated to enhance Raman signals more effectively since the enhancement of Raman signals that GO can generate is much less than that of noble metal NPs such as gold and silver NPs.

Hence, it can be concluded that a GO hybrid nanoparticle is highly useful not only for controlling stem cell behaviors, but also for monitoring their differentiation in a completely non-destructive and non-invasive manner, which expands the potential of GO hybrid nanoparticles as key materials for stem cell characterization.

## 6. Conclusions and Remarks

In this review, we highlighted a number of previously reported studies utilizing graphene or GO in various stem cell applications. Graphene and its derivatives, such as GO, graphene-based scaffolds, pure graphene hydrogels, and graphene hybrid nanoparticles, were reported to be effective in accelerating and guiding stem cell differentiation, controlling their growth and direction within 2D/3D environments, and, further, were shown to be useful in monitoring stem cell differentiation in an easy, precise, and non-destructive manner. Although the research has been promising, there are several issues that need to be clarified prior to the practical use of graphene derivatives in stem cell research, such as their potential cytotoxicity, adverse effects, and the speed of clearance of transplanted graphene. However, as recently reported by several studies investigating the *in vivo* biocompatibility of graphene and GO, which turned out to be safer than most other types of nanomaterials, it is highly likely that more intensive studies will be carried out in the near future to find proper ways to utilize graphene derivatives for stem cell applications, which may have an important role in accelerating the development of many different kinds of currently incurable diseases and disorders.
